# Emerging technologies for in-field plant virus detection: innovations and future directions

**DOI:** 10.1099/jgv.0.002182

**Published:** 2025-11-27

**Authors:** Subhankar Sahu, Rabah Boukherroub, Christophe Ritzenthaler, Sabine Szunerits

**Affiliations:** 1 Univ. Lille, CNRS, Univ. Polytechnique Hauts-de-France, UMR 8520 - IEMN, F-59000 Lille, France; 2 Institut de biologie moléculaire des plantes, CNRS, Université de Strasbourg, 67000 Strasbourg, France; 3 Laboratory for Life Sciences and Technology (LiST), Faculty of Medicine and Dentistry, Danube Private University (DPU), Viktor-Kaplan-Straße 2, Geb. E, 2700 Wiener Neustadt, Austria

**Keywords:** biosensor, diagnostic tool, phytopathology, plant virus, virus detection

## Abstract

Plant virus infections pose a substantial threat to crop quality and productivity, contributing to considerable economic losses in global agriculture annually. Traditionally, laboratories have widely adopted serological techniques, such as ELISA, and molecular methods, including quantitative PCR, for virus diagnostics. More recently, sophisticated next-generation sequencing approaches have been introduced to improve the efficiency and reliability of virus detection and identification. However, the development of sensitive, rapid and low-cost methods for the on-site detection, quantification and identification of plant viruses remains an ongoing challenge and is still in its early days. Point-of-care technologies have not fully realized their potential in agriculture due to numerous challenges, such as the elevated cost of development, lack of standardized validation and insufficient field testing. Therefore, future success depends on addressing these technical, economic and regulatory hurdles, as well as considering the specific user needs within the agricultural context. In this mini-review, recent advancements in biosensing for on-site plant virus monitoring, involving nanotechnology-based sensors, clustered regularly interspaced short palindromic repeats (CRISPR)-CRISPR-associated (Cas) systems, electrochemical and modern field-effect transistor-based sensors offering high sensitivity, speed and portability, are discussed. These technologies, when integrated with smartphone applications and/or machine learning modules, could enable real-time, field-deployable diagnostics for early disease management and sustainable agriculture. The aim is to raise awareness among plant virologists about this panel of emerging diagnostic concepts that could help improve current methods, ultimately facilitating the management of plant viral diseases.

**Impact Statement**

Professionals often rely on their own expertise and heuristic knowledge to suspect viral diseases in their crops and to manage their consequences. For more reliable assessment, plant samples can be collected and subjected to serological (i.e. ELISA) and/or nucleic acid amplification analyses (PCR, real-time quantitative PCR) in specialized laboratories by trained personnel using advanced equipment. While these techniques offer high performance, they are time-consuming, labour-intensive, rather costly and incompatible with on-field applications. The introduction of cost-effective biosensors with simple digital readouts that can deliver quantitative results within minutes would bridge the gap between visual assessment of disease symptoms and centralized lab testing. Recent advancements in materials science and electronics, along with the design of innovative, stable and high-affinity antibody mimetics, are set to propel the field of on-site plant virus detection forward. Future directions, involving refining portable, cost-effective kits and optimizing nanomaterial applications for multiplexing, will result in more robust, global surveillance platforms that will allow professionals to obtain real-time insight into the sanitary status of their crops and to take timely, appropriate actions.

## Introduction

Proactive disease management is gradually being acknowledged as a key strategy for strengthening the efficiency of healthcare in addressing chronic human diseases. Prophylactic measures also apply to plant viral disease management, where rapid and accurate identification of the causal agent is an essential measure when aiming at limiting viral spread by vectors or preventing the distribution and commercialization of infected plant material [1]. Plant virus infections cause estimated global economic losses exceeding $30 billion annually and account for nearly 50% of all plant diseases worldwide [2]. Despite their devastating impact and the clear need to prevent outbreaks, biosensing and diagnostic technologies for plant pathogens remain underdeveloped. Progress is slow due to the inherent complexity of plant disease detection, which requires rapid, sensitive and on-site methods that current practices often fail to provide. To be effective, any diagnostic procedure (Table 1) must be optimized for accuracy by determining both sensitivity (e.g. true-positive rate) and specificity (e.g. true-negative rate), while balancing numerous other factors, such as ease of use, time investment, cost, reliability, reproducibility, training requirement and throughput; this is especially true for plants, where mixed infections are more frequently encountered than in animals or humans [3,4]. Adding to this complexity, next-generation sequencing and metagenomics have considerably expanded our view and understanding of the huge diversity and rapid evolution that prevail among viruses, including phytoviruses [5,6].

**Table 1.** Examples of different sensing technologies and their features for plant virus detection discussed in this review. The ease in terms of sensor preparation and use is shown by a gradient of ‘+’. A similar gradient is shown for rapidity in terms of sensing readout time/response.

Technology	Virus	Plant	Sensitivity^*^	Rapidity†	Easiness	Ref.
Double-antibody sandwich LFIA	BBrMV	Banana	High 10 ng ml^−1^ coat protein, 1:20 dilution crude extract	++++ 10 min	++++	[19]
LAMP	TRV	Potato	High 15 pg RNA	+++ 20–45 min	+++	[24]
CRISPR-Cas12a and CRISPR-Cas13a/d implemented lateral flow assay (with and without RNA purification)	Tobacco mosaic virus (TMV) TEV PVX	Tobacco Potato	High Relative viral load of 10^−8^	+++ 40 min	+++	[5]
Plasmonic CRISPR-Cas12a assay coupled with PCR for the visual, colourimetric detection	GRBV	Grapevine	Very high 10 aM to 1 pM	++	++	[36]
ATR-mediated LSPR	ChiLCV	Chilli	Medium 1 µg ml⁻¹	+ ~100 min	+	[37]
MIP technology integrated with DPV	BPMV	Soybean	High 41 pg ml^−1^	++++ 2 min	++	[38]
Microfluidics-integrated electrochemical impedance biochips, LOC prototype	GFLV GLRaV-3	Grapevine	High 1:50 dilution for GFLV and 1:100 for GLRaV-3	++	++	[43]
Capacitive field-effect sensors, label-free electrostatic detection	TMV	Tobacco	Medium 1 µg ml^−1^ (0.025 nM)	+++ 30–60 min	++	[49]
Antibody-functionalized EGOFET	PPV	Plum	High 5 ng ml^−1^ to 50 µg ml^−1^	++	+	[50]
A-MSI device combined with machine learning for CBSD	CBSV UCBSV	Cassava	Medium 28 days post-inoculation	+++	+++	[52]
MinION and MinIT mobile sequencing devices (by Oxford Nanopore Technologies)	ACMV EACMV	Cassava	High	++ 180 min	+	[65]

*Sensitivity parameters are not uniform and are specified as mentioned in the original source, wherever available.

†Rapidity time values are included wherever available in the original sources.

ACMV, African cassava mosaic virus; ATR, attenuated total reflection; BBrMV, banana bract mosaic virus; BPMV, bean pod mottle virus; CBSD, Cassava brown streak disease; ChilLCV, chilli leaf curl virus; EACMV, East African cassava mosaic virus; EGOFET, electrolyte-gated organic field effect transistor; GFLV, grapevine fanleaf virus; GLRaV-3, grapevine leafroll-associated virus 3; GRBV, grapevine red blotch virus; LFA, lateral flow assay; PPV, Plum Pox Virus; PXV, Potato virus X; TEV, Tobacco etch virus; TMV, Tobacco mosaic virus; TRV, Tobacco rattle virus; UCBSV, Ugandan cassava brown streak virus.

**Fig. 1.**
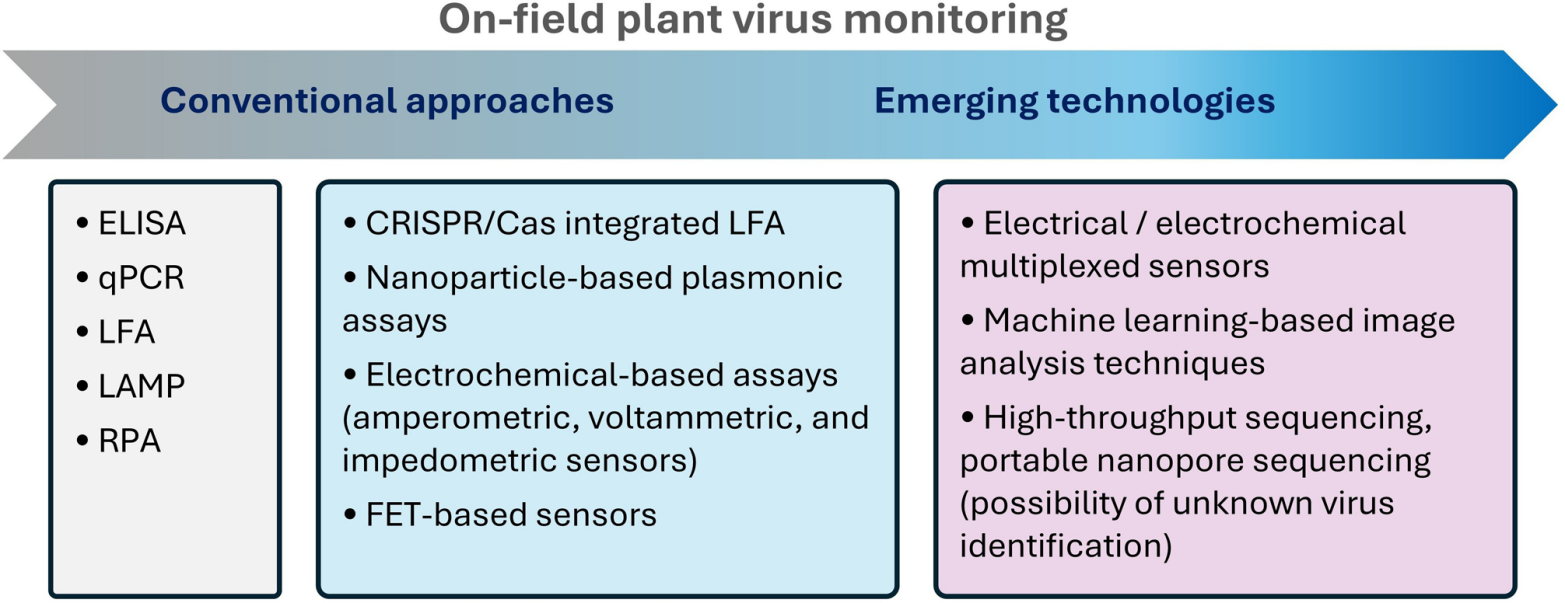
PoC diagnostic concepts applied to plant viruses: the evolution of emerging methods is discussed in comparison to the conventional approaches. LFA, lateral flow assay; RPA, recombinase polymerase amplification.

Although serological techniques, such as ELISA [essentially double-antibody sandwich (DAS)-ELISA], and molecular methods based on nucleic acid amplification [e.g. PCR and its derivative approaches, such as real-time PCR (RT-PCR)] for plant virus detection were first developed in the past century [7,10], they still remain the predominant methods for plant virus detection, owing to their high specificity, cost-effectiveness and relative ease of use. For example, BIOREBA AG, based in Switzerland, became the first company worldwide to produce and commercialize ELISA reagents for plant virus diagnostics in 1982, and its catalogue has expanded to PCR tests for the detection of a wide range of plant pathogens. Although PCR-based approaches are inherently more sensitive than serological methods [1,11], the adoption of quantitative PCR (qPCR) for plant virus diagnostics faces several challenges, including sample preparation (considering poor preparation can lead to false negatives, especially when inhibitors are present), the cost and equipment, which may be prohibitive for routine diagnostics, and the need for skilled personnel to perform the test and analyse the results. Despite the availability of these tools, there is a pressing need to develop plant virus sensing technologies that are sensitive, affordable and easy to use on-site, similar to those used for detecting animal and human viruses [12,13]. The point-of-care (PoC) application of such sensing tools ranges from portable organic electrochemical transistor (OECT)-based detection of Severe acute respiratory syndrome coronavirus 2 (SARS-CoV-2) Coronavirus disease 2019 (COVID-19) [14] to smartphone-based PoC [15] testing. This mini-review highlights emerging sensing technologies for on-site plant virus monitoring, including clustered regularly interspaced short palindromic repeats (CRISPR)/CRISPR-associated (Cas)-based systems, localized surface plasmon (LSP) and colourimetric methods, electrochemical assays and their multiplexed format, field-effect transistor (FET) sensors and high-throughput or nanopore sequencing (Fig. 1). These technologies are highly promising for building up future plant diagnostics; however, they still face numerous challenges in practical implementation and require further benchmarking to fully assess their effectiveness.

## Plant virus monitoring methods with on-site application potential

### Traditional portable technologies

**Fig. 2.**
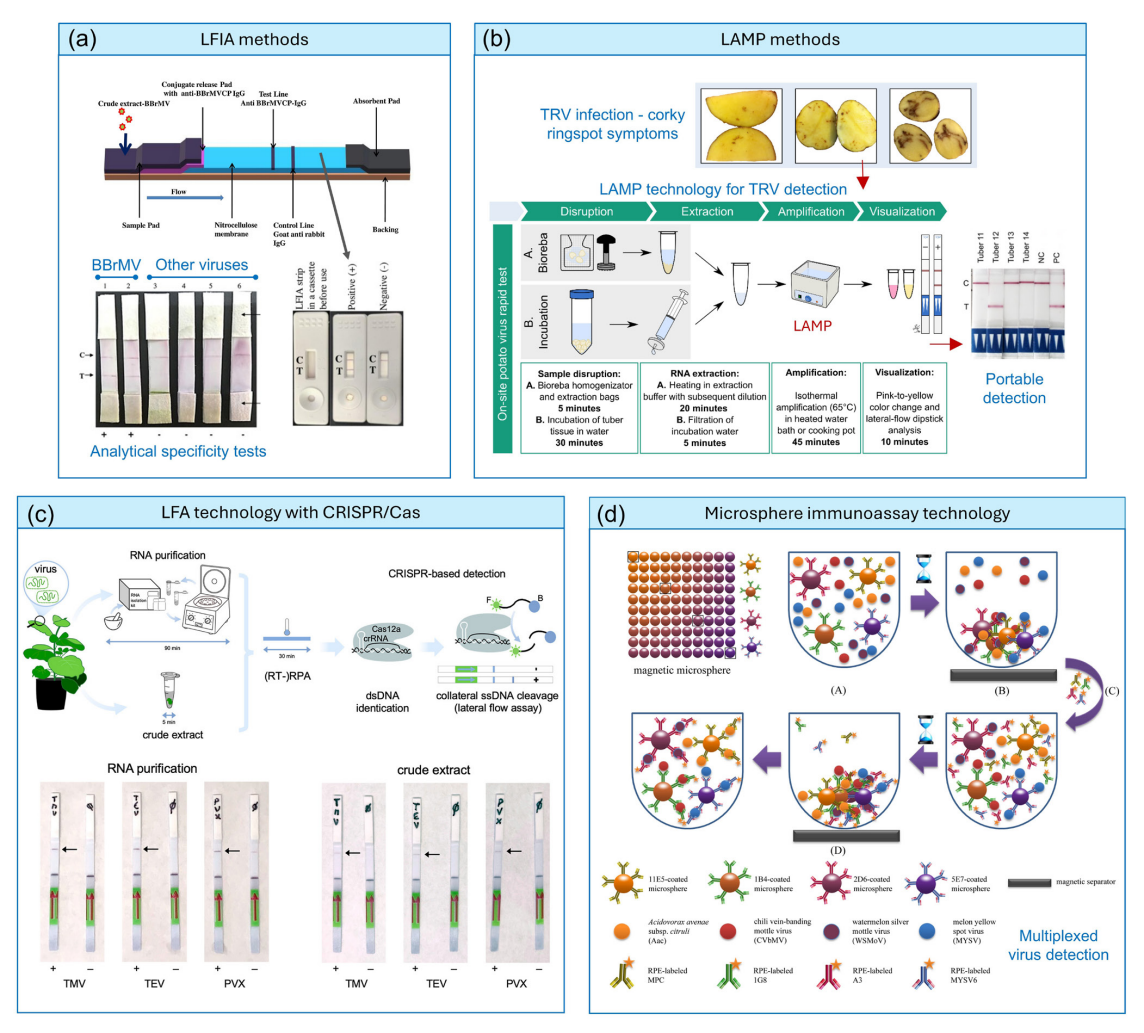
(**a**) Application of LFIA-based strips for BBrMV detection. The selectivity test for different viruses is shown (C: control line; T: test line) (reprinted with permission from Ref. [19]). (**b**) LAMP method for TRV detection (reproduced under the terms of the CC-BY 4.0 licence from Ref. [24]). (**c**) Schematic representation of field-deployable plant virus detection using CRISPR-Cas12a-based detection by a lateral flow assay. Two approaches were followed: with and without RNA purification. The arrows point to the positive test lines on the strips (reproduced under the terms of the CC-BY 4.0 licence from Ref [5]). (**d**) Magnetic microsphere immunoassay for multiplexed monitoring of plant pathogens (reproduced under the terms of the CC licence from Ref. [66]). (RT-)RPA: room temperature recombinase polymerase amplification. NC: negative control. PC: positive control.

Considering the evolution of plant virus sensing techniques, lateral flow immunoassay (LFIA) and loop-mediated isothermal amplification (LAMP) are among the earliest portable technologies to be developed and optimized. LFIA-based approaches are known for their simplicity, fast response and high cost-effectiveness [16]. The principle of LFIA dates from the late 1950s [17] and was initially developed for clinical diagnostics [18], before being adapted for plant virus detection in the late 1980s, offering rapid, on-site detection with minimal equipment. Over time, improvements in antibody technology and membrane materials have increased their sensitivity, specificity and multiplexing capabilities. Today, LFIA strips are widely used for quick field diagnosis of plant viruses, supporting effective crop disease management. One of the recent examples is the work by Selvarajan *et al.* [19]*,* utilizing a DAS format LFIA for monitoring the banana bract mosaic virus (BBrMV) (Fig. 2a). The test recorded a detection limit of 10 ng mL^-1^ for the expressed recombinant BBrMV coat protein and a 1:20 dilution of crude extract from BBrMV-infected samples. The authors also validated the real-time application of such technology by randomly collecting field samples (114 banana leaf samples), with high diagnostic sensitivity (99.04%) and specificity (100%). A similar strategy was employed for the ultrasensitive and simultaneous detection of two important sugarcane viruses, sugarcane mosaic virus (SCMV) and sugarcane streak mosaic virus (SCSMV), using a cysteamine–gold nanoparticles (GNPs)-implemented LFIA platform for enhanced detection [20]. A linear detection range of 10 ng ml^−1^ to 10 µg ml^−1^ was achieved, with the detection limit lowered to 10 pg ml^−1^ upon nanoparticle-induced signal enhancement, matching RT-PCR-level sensitivity.

As an alternative to LFIA, nucleic acid-based diagnostic technologies such as LAMP were proposed in the early 2000s [21], offering several advantages over RT-qPCR for plant virus diagnosis [22]. LAMP does not require sophisticated equipment, such as thermal cyclers, is extremely robust against common inhibitors, is compatible with portable devices (e.g. (lateral flow immunoassay) LFIA-based strips, smartphone-based readers, microfluidic chips, etc.) and can be tailored to produce visual readouts as well [23]. Edgü *et al*. [24] proposed a colourimetric LAMP for the detection of Tobacco rattle virus (TRV), causing corky ringspot and being a significant threat to potato cultivation, without any RNA purification step, in a lateral flow dipstick (LFD) analysis format (Fig. 2b). The LAMP–LFD exhibited 89% sensitivity and 100% specificity, delivering precision and reliability similar to RT-PCR. Other examples include the use of LAMP for on-site detection of tomato mosaic virus [25], cassava brown streak virus (CBSV) and Ugandan cassava brown streak virus (UCBSV) [26], tomato leaf curl New Delhi virus [27] and sweet potato viruses by an ultra-portable, pocket-sized device [28]. Further dedicated discussions on LAMP or LFIA can be found in references [16,29,32].

### Potential of multiplexed virus detection

Multiplexed sensing – simultaneously detecting and identifying multiple viruses or virus strains in a single assay – significantly reduces the time and cost of analysis. Several sensing technologies based on CRISPR and Cas proteins, such as Cas9, Cas12 and Cas13, have been adapted recently for rapid, sensitive and specific detection of plant viruses, offering potential improvements over traditional diagnostic methods in terms of speed, simplicity, multiple virus detection capabilities and field applicability. Combined with isothermal amplification approaches, CRISPR-Cas systems in PoC format can detect plant pathogens with extreme specificity and sensitivity [31]. As a recent example, Marqués *et al*. [5] successfully exploited CRISPR-Cas12a and CRISPR-Cas13a/d systems to detect tobacco mosaic virus, tobacco etch virus (TEV) and potato virus X (PVX) in *Nicotiana benthamiana*. In particular, by combining CRISPR-Cas12a-based detection with PCR or isothermal amplification and lateral flow assay (Fig. 2c), they managed to directly detect TMV, TEV and PVX. The researchers also exploited amplification-free CRISPR-Cas13a-based detection by fluorescence readout and showed that it scaled with the viral load in the plant. These methods enable viral diagnostics to be completed within ~30 min, making them potentially suitable for field-deployable applications.

While techniques such as microarrays can screen many samples at once, microsphere technologies, such as the Luminex xMAP (Multi-Analyte Profiling), also offer an advanced alternative as a multiple virus detection platform. This versatile technology uses magnetic, colour-coded microspheres (beads) coated with specific antibodies to capture viruses (Fig. 2d), allowing the simultaneous detection of up to 50 targets in a single sample [13,14]. Additionally, the use of magnetic beads allows efficient removal of excess sample compounds, thereby lowering background noise while improving specificity. Another key advantage of xMAP technology is its improved limit of detection (LoD), along with considerable cost savings due to reduced labour and consumables requirements, with an assay completion time of ~3–4 h. Nevertheless, the main drawbacks of this technology include cross-reactivity and the need for rigorous optimizations of antibody pairs, blocking buffers and labelling conditions to avoid high background.

### Nanoparticle-based colourimetric and plasmonic methods

**Fig. 3.**
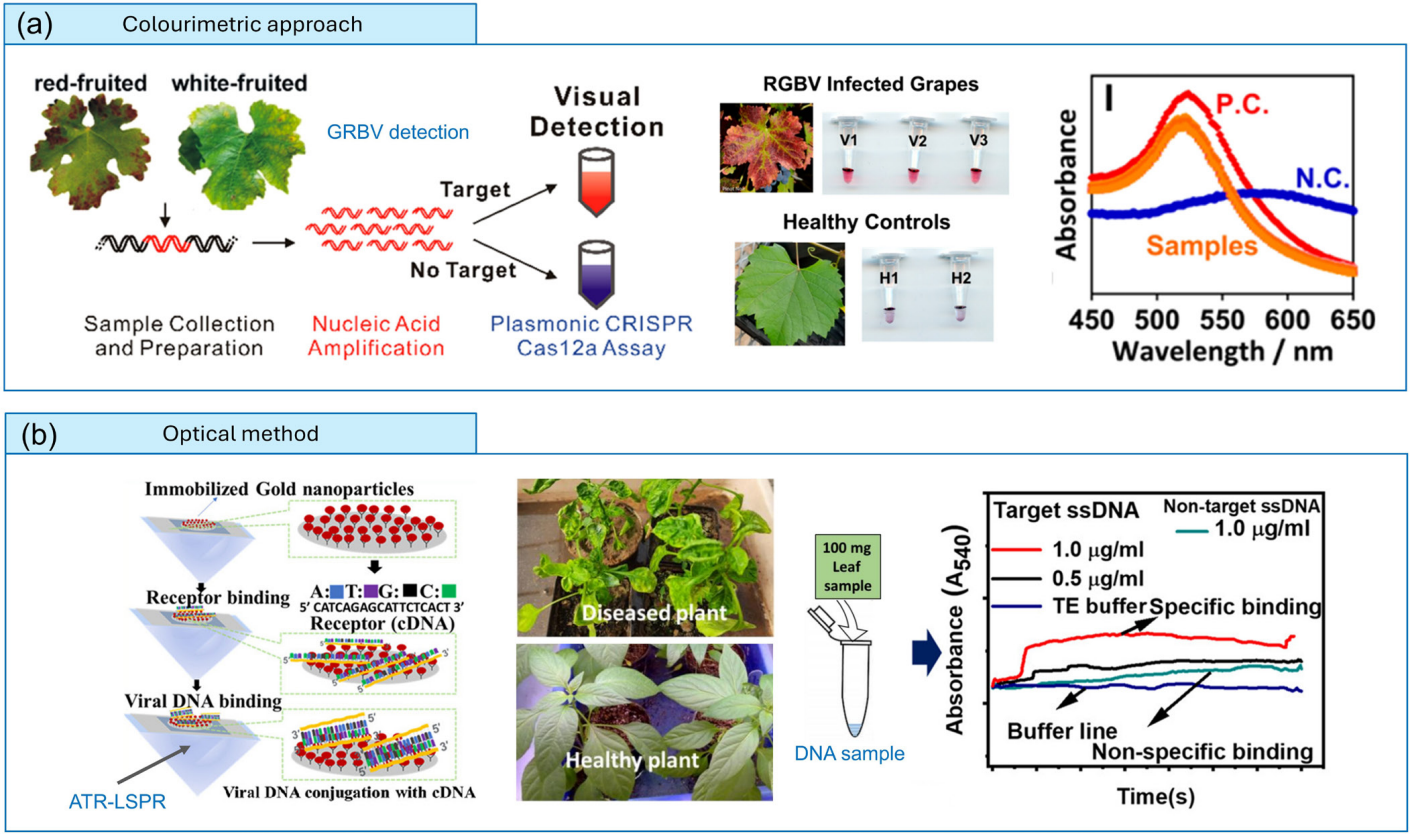
Optical techniques for plant virus monitoring. (**a**) Naked-eye detection of GRBV by a plasmonic CRISPR-Cas12a assay. This implements DNA-functionalized GNPs, and in the absence of the virus, the sensing solution mixture changes colour from red to blue (reprinted with permission from Ref. [36]). (**b**) In-house fabricated ATR-mediated LSPR-based optical platform for ChiLCV monitoring (reproduced under the terms of the CC-BY-NC-ND 4.0 licence from Ref. [37]). ATR, attenuated total reflection. N.C.: negative control, P.C.: positive control, TE: Tris–EDTA buffer.

CRISPR-Cas12a-based technology, widely used for virus detection [33,35], has recently been applied for the visual detection of emerging viruses, such as the grapevine red blotch virus (GRBV). Li *et al*. [36] demonstrated that DNA-functionalized GNPs can serve as molecular probes for reliable, naked-eye colourimetric detection of GRBV at concentrations as low as the picomolar level (Fig. 3a). This detection strategy relies on the conventional aggregation kinetics of GNPs, where the colour shifts from blue to red in the presence of the viral marker. Despite their sensitivity and simplicity, these methodologies often face challenges related to probe stability, uncontrolled aggregation and the need for sample pre-treatment.

In a separate study, an attenuated total reflection-mediated LSP resonance (LSPR)-based optical platform was developed for monitoring chilli leaf curl virus (ChiLCV) (Fig. 3b). In this work, the ssDNA of ChiLCV was detected using its cDNA strand over the LSPR probe surface [37]. Although such platforms are still in their early stages of development, achieving an LoD of only 1 µg ml⁻¹, they hold promising potential as optical instruments for virus monitoring. At present, these technologies have limited field applications and limited real plant sap usage due to shortcomings of plasmonic platforms, particularly those using gold nanoparticles because they are highly sensitive to changes in ambient refractive index, which can be influenced by temperature or humidity.

### Emerging electrochemical technologies

#### Differential pulse voltammetry and electrochemical impedance spectroscopy-based methods

**Fig. 4.**
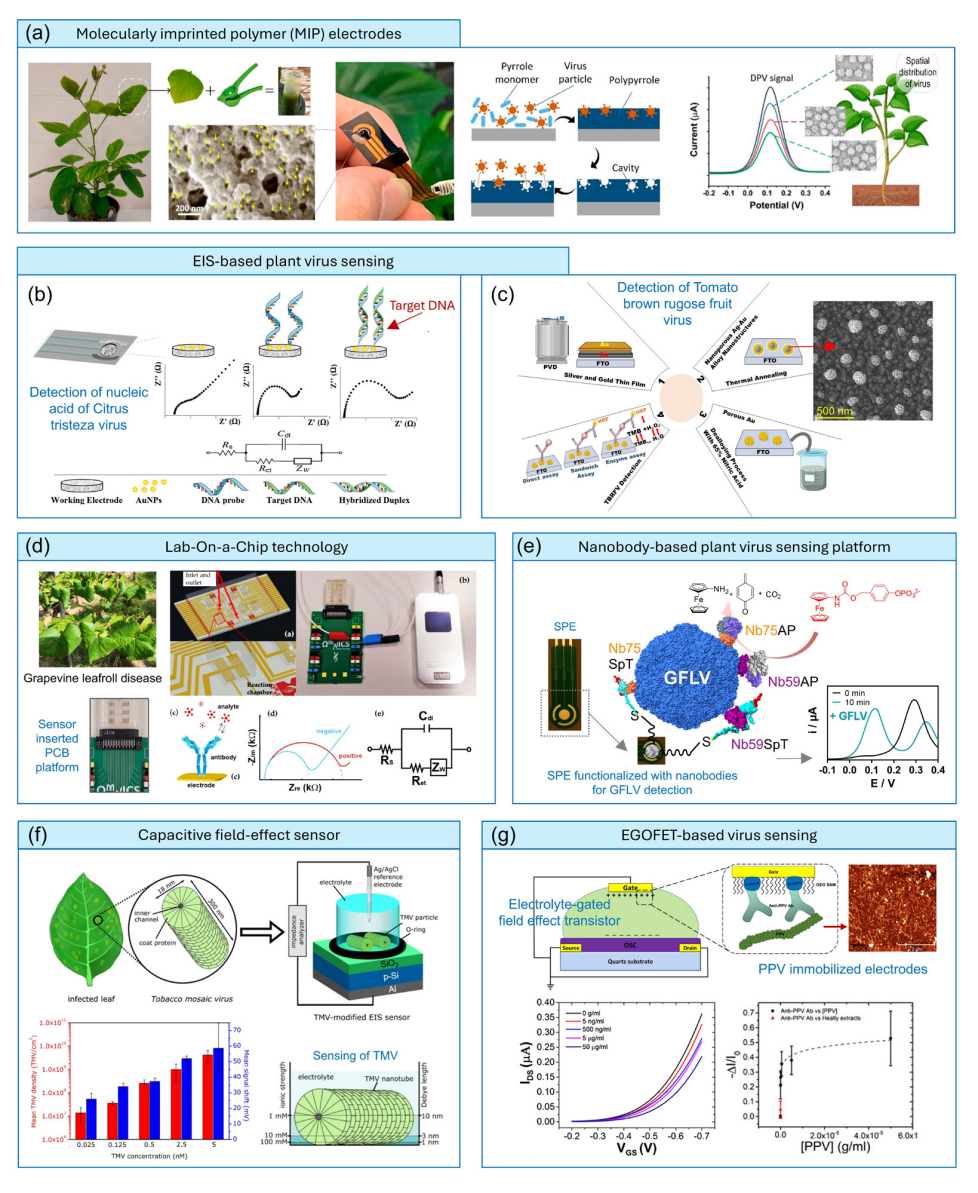
(**a**) Sensing of BPMV by molecularly imprinted polypyrrole electrodes. Virus-specific nanocavities are used for BPMV quantification by monitoring the DPV response (reprinted with permission from Ref. [38]). (**b**) AuNP-modified electrodes for CTV by EIS measurements (reproduced under the terms of the CC-BY licence from Ref [67]). (**c**) EIS-based electrochemical and sandwich immunoassay for TBRFV detection (adapted under the terms of the CC-BY 4.0 licence from Ref. [41]). (**d**) An LOC prototype for GFLV and GLRaV-3 detection, which can be operated with a portable potentiostat. A multiplexed microchamber is interfaced with an antibody-functionalized electrode array for virus sensing (reproduced under the terms of the CC-BY 4.0 licence from Ref. [43]). (**e**) Electrochemical sensor for GFLV using a screen-printed gold electrode modified with nanobodies for GFLV for virus capture and alkaline phosphatase-modified nanobodies (Nb-GFLV-ALP) for detection. Readout is via DPV using 4-[(ferrocenylcarbamoyl)oxy]phenyl disodium phosphate, an enzymatic substrate [45]. (**f**) Sensing of TMV by an FET-based approach. An illustration of a TMV nanotube with regularly ordered coat proteins, along with the measurement setup for a TMV-modified EIS sensor, is shown (reproduced under the terms of the CC-BY 4.0 licence from Ref. [49]). (**g**) Application of the EGOFET strategy for PPV. The gate electrode is suitably functionalized with anti-PPV antibodies for virus binding, and detection is achieved through a change in drain-current response from the transfer characteristics curve (reprinted with permission from Ref. [50]). SPE, screen-printed electrode. Au NPs: gold nanoparticles, DPV: differential pulse voltammetry, Ab: antibody, FTO: fluorine-doped tin oxide, Au: gold, PCB: printed circuit bord, Ag/AgCl: silver/silver chloride, p-Si: p-doped silicon, IDS: drain-source current, VGS: gate-source voltage

Electrochemical biosensing approaches are known for their high sensing accuracy, rapid response times, operational simplicity and suitability for on-site or PoC applications. In the domain of plant virus sensing, electrochemical approaches, including differential pulse voltammetry (DPV) and electrochemical impedance spectroscopy (EIS), are among the most explored methods, and, very recently, efforts on transistor-type approaches have also been investigated. Some of the research regarding these methods is discussed in the following section. The application of molecularly imprinted polymer (MIP) technology was explored by Singh *et al*. [38] for the ultrasensitive detection of bean pod mottle virus (BPMV) in soybean plants (Fig. 4a). Using porous polypyrrole, they could produce BPMV-specific nanocavities in the electrode, and by DPV, these biosensors could easily surpass conventional techniques such as ELISA and RT-PCR-based methods, achieving an LoD of 41 pg ml^−1^. Besides detecting infected plants from non-infected ones within 2 min, the sensor could also provide the spatial distribution of BPMV levels across different leaves. A MIP-based approach was very recently designed for TMV detection as well [39]. For this, a MIP monoclonal antibody targeting TMV was integrated into a paper-based strip, and the virus presence was monitored under either visible or ultraviolet light irradation, or fluorescence spectroscopy, with an LoD of 4.8 fM. This prototype costs only ~8.6 cents and, once benchmarked against real samples, could be a highly promising option for crop virus diagnosis.

The use of AuNP-modified electrodes for Citrus tristeza virus (CTV) is an example of the application of EIS measurements using Fe(CN_6_)^4−^/Fe(CN_6_)^3−^ redox system [40]. CTV has contributed to elevated mortality rates worldwide, particularly in regions where citrus seedlings are grafted onto sour orange rootstocks. The AuNP-functionalized screen printed carbon electrode (SPCE) could monitor CTV-related nucleic acid between 0.1 and 10 µM through employing a label-free impedance approach (Fig. 4b). Another similar EIS-based electrochemical sandwich immunoassay, using a nanoporous gold electrode, was reported by Rezaei *et al*. [41] for the detection of tomato brown rugose fruit virus (TBRFV) with attomolar sensitivity: 65.14 aM (1.14 fg ml^−1^) in the case of the direct assay and 60.57 aM (1.06 fg ml^−1^) for the enzyme-based sandwich assay (Fig. 4c).

Simultaneous detection of multiple viruses is particularly important in plant pathology, especially for economically significant crops such as grapevine, which hosts over 100 identified virus species [42]. Buja *et al.* [43] have reported microfluidics-integrated EIS biochips for simultaneously monitoring grapevine leafroll-associated virus 3 (GLRaV-3) and grapevine fanleaf virus (GFLV) (Fig. 4d). In order to circumvent the disadvantages of ELISA-based approaches, they developed a lab-on-a-chip (LOC) prototype that can be operated with a portable potentiostat. This LOC platform features an array of interdigitated microelectrodes functionalized with antibodies specific to GLRaV-3 and GFLV, achieving a sensitivity that exceeds ELISA by 20 times. Once these technologies are validated through rigorous field-sample testing, they may prove to be highly pertinent for on-field virus diagnostics.

Nanobodies (Nbs) are another type of interesting bioreceptors for specific target virus detection and are often considered a better alternative to antibodies, because of their small size (12–15 kDa), ease of production, low cost and high affinity [44]. The team of Ritzenthaler has recently engineered Nbs, derived from camelids specifically targeting GFLV [45,46], and developed a double-Nb sandwich electrochemical assay using DPV. In this work, similar to the DAS-ELISA, a set of capture Nbs immobilized on gold electrodes was employed for the binding/capturing, and the same set of Nbs genetically fused to recombinant alkaline phosphatase was exploited for GFLV detection in both herbaceous hosts and grapevines (Fig. 4e). Using this approach, an LoD comparable to that of conventional DAS-ELISA used for certification was achieved, with the advantage of field-based prospects and a readout time of ~30 min [45].

#### FET-based sensors

In the rapidly evolving field of bioelectronic sensors, FETs are recognized as one of the most advanced transducer platforms [47]. FETs are essentially three-terminal (source: S, drain: D and gate: G) semiconductor devices in which the gate-induced electric fields regulate the channel carrier density and, therefore, the source and drain current. In bio-FETs, any underlying sensing event (mostly in the channel region) alters the surface potential, resulting in proportional changes in the output current/voltage. The major advantages of this type of actuator are high sensitivity and the absence of the need for any redox mediator, as commonly used in amperometric approaches [48]. Their application for virus detection in animals [e.g. SARS-CoV-2, H1N1 (Influenza A virus subtype H1N1, where H stands for hemagglutinin and N for neuraminidases), etc.] [48] has been proven to be highly reliable. Nevertheless, very few examples exist in this regard for plant viruses. Jablonski *et al.* [49] investigated the use of capacitive field-effect sensors for the detection of TMV as a model analyte (Fig. 4f). The field-effect (silicon dioxide) SiO_2_-gate electrolyte–insulator–semiconductor sensor successfully identified TMV particles in solution at an ultra-low concentration of 0.025 nM (0.001 mg ml^−1^). Although the demonstrated technology is a proof-of-concept for label-free TMV detection, it holds high potential to be extended to other plant pathogens.

Bioelectric sensors based on electrolyte-gated organic FETs (EGOFETs) have also been reported recently for the specific and sensitive detection of plant viruses. For instance, in a 2019 study by Berto *et al*. [50] (Fig. 4g), anti-plum pox virus (PPV) antibodies were immobilized in a controlled orientation on a gold gate using a monolayer of protein G. The EGOFET’s ability to selectively detect PPV was assessed by incubating the gate *ex situ* and recording the transfer characteristics. The addition of PPV resulted in a decrease in the slope of the linear region of the transfer curve, primarily due to variations in transconductance, with only minor alterations in the threshold voltage. This EGOFET achieved a sub-nanogram per millilitre LoD for PPV in plant extracts, with a dynamic range spanning from 5 ng ml^−1^ to 50 µg ml^−1^. Overall, the rapid progress in EGOFET and OECT-based technologies for human virus detection [14] suggests that these approaches will soon be adapted for plant virus diagnostics. Notably, studies have recently begun applying OECTs for applications such as stress monitoring [51], leveraging state-of-the-art ion-selective-OECT biosensing technology.

### Machine learning-based methods

**Fig. 5.**
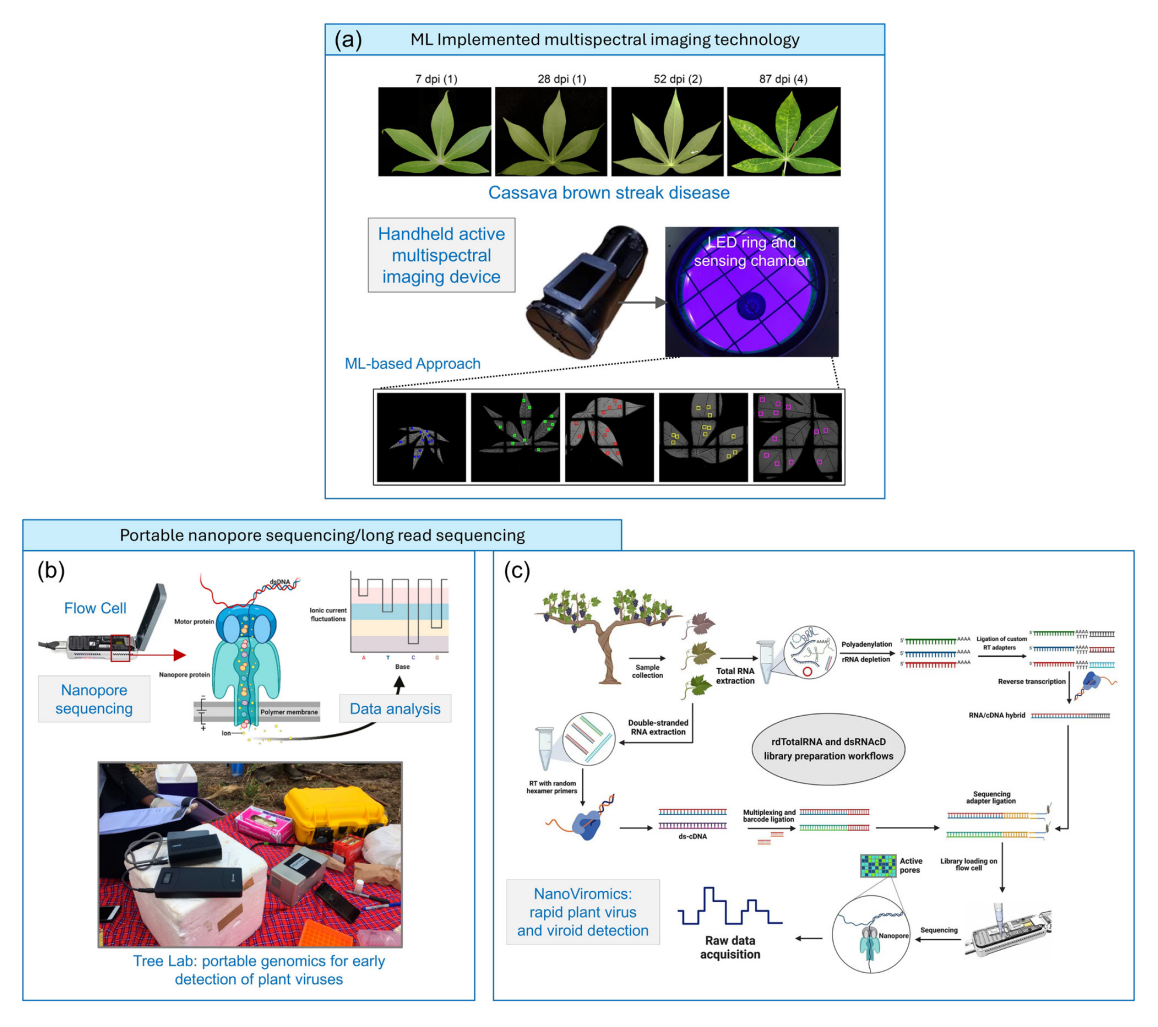
(**a**) Handheld A-MSI device for detecting CBSV. Leaves at different infection time points (dpi) are shown. The prototype device, along with the snapshots of the leaves, is represented (reproduced under the terms of the CC-BY 4.0 licence from Ref. [52]). (**b**) Nanopore sequencing technology for on-field detection of plant viruses. The nucleic acid material passes through the nanopore, and the resulting electrical response is deconvoluted and base-called to retrieve the sequence (image adapted from Ref [68]). Image of the Tree Lab – a portable lab for cassava DNA virus detection using Nanopore MinION Sequencing (Oxford Nanopore Technologies) (reproduced under the terms of the CC-BY 4.0 licence from Ref. [65]). (**c**) dsRNA-based nanopore sequencing pipeline for plant virology diagnostics in grapevine and comparison of critical factors such as speed, sensitivity and cost (reproduced under the terms of CC-BY licence from Ref. [58]). dpi, days post-inoculation; ML, machine learning.

Recently, there has also been recognized interest in the community in designing handheld imaging devices for plant viruses, particularly due to their portability and high field application relevance. For example, Peng *et al*. [52] fabricated an active multispectral imaging (A-MSI) device, which, when combined with an underlying machine learning algorithm, can be used for the early detection of cassava brown streak disease caused by CBSV (Fig. 5a). This A-MSI approach is more advantageous than conventional multispectral imaging techniques due to its enhanced signal-to-noise ratio, and it can reliably distinguish between the diseased and healthy cassava plants as early as 28 days post-inoculation. In this non-invasive technique, the device captures images of the leaves at 14 different wavelengths (from visible to near-infrared), after which spectral and spatial features are extracted from small patches and fed into a Support Vector Machine (SVM)-based learning model for classification. From our perspective, considering the evolution of artificial intelligence in image/data analysis [53], this type of method is soon expected to transcend to other plant viruses as well, especially where symptoms are seen early.

## Conclusions and future outlook

ELISA and nucleic acid-based detection methods remain the most widely used techniques for routine plant virus diagnosis, due to their sensitivity, reliability and commercial availability. Having said that, biosensor technology has also advanced significantly in recent years, driven by its potential to efficiently detect plant viruses. Despite this progress, a significant gap persists between the developed technologies and their practical application, highlighting challenges in accessibility and usability. Key issues, such as sensor stability, biofouling of sensing transducers, surface passivation due to protein adsorption from the complex plant extracts, reproducible immobilization of bioreceptors, upscaling and commercialization, remain inadequately addressed. The complex matrix of plant extract often requires the implementation of specific pre-treatment processes before sensing. Without resolving these challenges, sensors cannot achieve the sensitivity and reliability required for accurate field-based diagnostics.

While the aforementioned techniques require prior knowledge of the pathogen to be detected, high-throughput sequencing (HTS) has transformed plant virus diagnostics by enabling the simultaneous detection of both known and unknown viruses in an agnostic manner, allowing hypothesis-free testing of plant samples. The advantages and disadvantages of HTS for phytovirus diagnostics, disease management and quarantine measures are greatly outlined in some recent review articles [54,57]. While HTS is believed to become the future standard in plant virology, it currently lacks the ability to be employed as a portable diagnostic for field analysis and needs skilled bioinformaticians. In parallel, portable long-read direct sequencing technologies (especially from Oxford Nanopore Technologies – MinION or PromethION) have huge potential in the future for direct on-field virus monitoring and can be more relevant when any specific knowledge about the target virus is not available or in a mixed infection. One such plant virus sensing platform is ‘Tree Lab’, in which, by employing the MinION and MinIT mobile sequencing devices, developed by Oxford Nanopore Technologies, a group of scientists have been able to develop a platform integrating portable genomics for early detection of plant viruses and pests in Sub-Saharan Africa (Fig. 5b) [65]. This identifies cassava DNA viruses on the farms in Tanzania, Uganda and Kenya using the portable DNA sequencer technology, wherein the sample collection and diagnosis in the field can be achieved within 3 h. In another work, Javaran *et al*. [58] designed a dsRNA-based nanopore sequencing pipeline (referred to as ‘NanoViromics’) for the detection of viruses in grapevines (Fig. 5c). They demonstrated that the dsRNA-implemented direct Oxford nanopore sequencing protocol outperformed traditional total RNA methods in most diagnostic metrics and can be implemented in routine plant health monitoring programmes. Nevertheless, due to the high cost and requirement for ultra-pure virus nucleic acid material, on-field application of such technology is currently limited. The nanopore sequencing approach has already been proven to be useful for the portable detection of animal viruses [59,61], and owing to the fast progress in low-cost portable electronics and barcoding techniques, it is expected to soon transcend to high-scale field application.

Simultaneously, nanomaterials have demonstrated potential in mitigating anti-fouling issues and enhancing sensor sensitivity, particularly when combined with high-affinity bioreceptors and innovative biofunctionalization strategies [62,63]. Interfacing capillary-based prefiltering steps or microfluidic platforms could also provide far more accurate solutions for in-field sensing. Furthermore, integrating technology-assisted decision-making tools will require collecting high-quality data from extensive areas using individual sensors. The integration of remote monitoring through wireless sensor networks and internet of things (IoT)-based systems represents the next major advancement in this field [64]. In conclusion, the sensor research community is actively addressing these limitations, with ongoing efforts focused on optimizing real-sample performance, enhancing portability and improving field applicability. Bridging the gap between advanced biosensor technologies and their practical implementation will be crucial for transforming plant virus diagnostics and disease management.

## Abbreviations

A-MSI: active multispectral imaging
Au: gold
BBrMV: banana bract mosaic virus
BPMV: bean pod mottle virus
Cas: CRISPR-associated
CBSV: cassava brown streak virus
ChiLCV: chilli leaf curl virus
COVID-19: coronavirus disease 2019
CRISPR: clustered regularly interspaced short palindromic repeats
CTV: citrus tristeza virus
DAS: double-antibody sandwich
DPV: differential pulse voltammetry
EGOFET: electrolyte-gated organic field-effect transistor
EIS: electrochemical impedance spectroscopy
FET: field-effect transistor
GFLV: grapevine fanleaf virus
GLRaV-3: grapevine leafroll-associated virus 3
GNPs: gold nanoparticles
GRBV: grapevine red blotch virus
H1N1: influenza A virus subtype H1N1
HTS: high-throughput sequencing
IoT: internet of things
LAMP: loop-mediated isothermal amplification
LFD: lateral flow dipstick
LFIA: lateral flow immunoassay
LFIA: lateral flow immunoassay
LOC: lab-on-a-chip
LoD: limit of detection
LSP: localized surface plasmon
LSPR: localized surface plasmon resonance
MIP: molecularly imprinted polymer
Nbs: nanobodies
OECT: organic electrochemical transistor
PoC: point-of-care
PPV: plum pox virus
PVX: potato virus X
qPCR: quantitative PCR
RT-PCR: real-time PCR
SARS-CoV-2: severe acute respiratory syndrome coronavirus 2
SCMV: sugarcane mosaic virus
SCSMV: sugarcane streak mosaic virus
SiO2: silicon dioxide
TBRFV: tomato brown rugose fruit virus
TEV: tobacco etch virus
TRV: tobacco rattle virus
UCBSV: Ugandan cassava brown streak virus

## References

[1] Rubio L, Galipienso L, Ferriol I (2020). Detection of plant viruses and disease management: relevance of genetic diversity and evolution. Front Plant Sci.

[2] Hilaire J, Tindale S, Jones G, Pingarron-Cardenas G, Bačnik K (2022). Risk perception associated with an emerging agri-food risk in Europe: plant viruses in agriculture. Agric Food Secur.

[3] Singhal P, Nabi SU, Yadav MK, Dubey A (2021). Mixed infection of plant viruses: diagnostics, interactions and impact on host. *J Plant Dis Prot*.

[4] Syller J (2012). Facilitative and antagonistic interactions between plant viruses in mixed infections. Mol Plant Pathol.

[5] Marqués M-C, Sánchez-Vicente J, Ruiz R, Montagud-Martínez R, Márquez-Costa R (2022). Diagnostics of infections produced by the plant viruses TMV, TEV, and PVX with CRISPR-Cas12 and CRISPR-Cas13. ACS Synth Biol.

[6] Riyaz SM, Jesse DMI, Kathiravan K (2021). In Plant Virus-Host Interaction. Plant Virus-Host Interaction.

[7] Clark MF, Adams AN (1977). Characteristics of the microplate method of enzyme-linked immunosorbent assay for the detection of plant viruses. J Gen Virol.

[8] Vunsh R, Rosner A, Stein A (1990). The use of the polymerase chain reaction (PCR) for the detection of bean yellow mosaic virus in gladiolus. Ann Appl Biol.

[9] Robertson NL, French R, Gray SM (1991). Use of group-specific primers and the polymerase chain reaction for the detection and identification of luteoviruses. J Gen Virol.

[10] López-Moya JJ, Cubero J, López-Abella D, Díaz-Ruíz JR (1992). Detection of cauliflower mosaic virus (CaMV) in single aphids by the polymerase chain reaction (PCR). J Virol Methods.

[11] Torre C, Agüero J, Gómez‐Aix C, Aranda MA (2020). Comparison of DAS‐ELISA and qRT‐PCR for the detection of cucurbit viruses in seeds. Ann Appl Biol.

[12] Marrazza G, Ramalingam M, Jaisankar A, Cheng L, Selvolini G (2024). Advancements and emerging technologies in biosensors for rapid and accurate virus detection. TrAC Trend Anal Chem.

[13] Nguyen HA, Anh Thi NP, Thien Trang NP, Ho T-T, Trinh TND (2024). Recent advances in biosensors for screening plant pathogens. Anal Methods.

[14] Liu H, Yang A, Song J, Wang N, Lam P (2021). Ultrafast, sensitive, and portable detection of COVID-19 IgG using flexible organic electrochemical transistors. Sci Adv.

[15] Xiao M, Tian F, Liu X, Zhou Q, Pan J (2022). Virus detection: from state‐of‐the‐art laboratories to smartphone‐based point‐of‐care testing. Adv Sci.

[16] Kalimuthu K, Arivalagan J, Mohan M, Samuel Selvan Christyraj JR, Arockiaraj J (2022). Point of care diagnosis of plant virus: current trends and prospects. Mol Cell Probes.

[17] Li G, Li Q, Wang X, Liu X, Zhang Y (2023). Lateral flow immunoassays for antigens, antibodies and haptens detection. Int J Biol Macromol.

[18] Andryukov BG (2020). Six decades of lateral flow immunoassay: from determining metabolic markers to diagnosing COVID-19. *AIMS Microbiol*.

[19] Selvarajan R, Kanichelvam PS, Balasubramanian V, Sethurama Subramanian S (2020). A rapid and sensitive lateral flow immunoassay (LFIA) test for the on-site detection of banana bract mosaic virus in banana plants. J Virol Methods.

[20] Thangavelu RM, Kadirvel N, Balasubramaniam P, Viswanathan R (2022). Ultrasensitive nano-gold labelled, duplex lateral flow immunochromatographic assay for early detection of sugarcane mosaic viruses. Sci Rep.

[21] Notomi T, Okayama H, Masubuchi H, Yonekawa T, Watanabe K (2000). Loop-mediated isothermal amplification of DNA. Nucleic Acids Res.

[22] Panno S, Matić S, Tiberini A, Caruso AG, Bella P (2020). Loop mediated isothermal amplification: principles and applications in plant virology. *Plants (Basel*).

[23] Yadav A, Yadav K (2025). Portable solutions for plant pathogen diagnostics: development, usage, and future potential. Front Microbiol.

[24] Edgü G, Freund LJ, Hartje S, Tacke E, Hofferbert H-R (2020). Fast, precise, and reliable multiplex detection of potato viruses by loop-mediated isothermal amplification. Int J Mol Sci.

[25] Kirasi PM, Ateka EM, Avedi EK, Yegon HK, Wanjala BW (2024). A reverse transcription loop-mediated isothermal amplification assay for quick detection of tomato mosaic virus. PLoS One.

[26] Tomlinson JA, Ostoja-Starzewska S, Adams IP, Miano DW, Abidrabo P (2013). Loop-mediated isothermal amplification for rapid detection of the causal agents of cassava brown streak disease. J Virol Methods.

[27] Caruso AG, Ragona A, Bertacca S, Montoya MAM, Panno S (2023). Development of an in-field real-time LAMP assay for rapid detection of tomato leaf curl New Delhi virus. *Plants*.

[28] Fuentes S, Ogero K, Perez A, Kreuz JF (2025). Evaluation of an ultra-portable pocket-sized device for running loop mediated isothermal amplification (LAMP) assays for rapid detection of sweetpotato viruses. VeriXiv.

[29] Garg N, Ahmad FJ, Kar S (2022). Recent advances in loop-mediated isothermal amplification (LAMP) for rapid and efficient detection of pathogens. Curr Res Microb Sci.

[30] Fu R, Sha Y, Xu X, Liu S-B (2024). Advancements in the loop-mediated isothermal amplification technique for the rapid detection of plant viruses in various crops. Physiol Mol Plant Pathol.

[31] Yang L, Sun Y, Sun L, Wang Z, Feng J (2025). Application of loop-mediated isothermal amplification in plant pathogen detection. Phytopathology.

[32] Patel R, Mitra B, Vinchurkar M, Adami A, Patkar R (2022). A review of recent advances in plant-pathogen detection systems. Heliyon.

[33] Mahas A, Hassan N, Aman R, Marsic T, Wang Q (2021). LAMP-coupled CRISPR-Cas12a module for rapid and sensitive detection of plant DNA viruses. Viruses.

[34] Jiao J, Kong K, Han J, Song S, Bai T (2021). Field detection of multiple RNA viruses/viroids in apple using a CRISPR/Cas12a-based visual assay. Plant Biotechnol J.

[35] Aman R, Mahas A, Marsic T, Hassan N, Mahfouz MM (2020). Efficient, rapid, and sensitive detection of plant RNA viruses with one-pot RT-RPA-CRISPR/Cas12a assay. Front Microbiol.

[36] Li Y, Mansour H, Wang T, Poojari S, Li F (2019). Naked-eye detection of grapevine red-blotch viral infection using a plasmonic CRISPR Cas12a assay. Anal Chem.

[37] Das S, Agarwal DK, Mandal B, Rao VR, Kundu T (2021). Detection of the chilli leaf curl virus using an attenuated total reflection-mediated localized surface-plasmon-resonance-based optical platform. ACS Omega.

[38] Singh N, Khan RR, Xu W, Whitham SA, Dong L (2023). Plant virus sensor for the rapid detection of bean pod mottle virus using virus-specific nanocavities. ACS Sens.

[39] Gong H, Pang T, Yang X, Chen F, Jiang N (2025). Rapid visual detection of tobacco mosaic virus using a portable paper-based molecularly imprinted sensor. Sens Actuators B Chem.

[40] Khater M, de la Escosura-Muñiz A, Quesada-González D, Merkoçi A (2019). Electrochemical detection of plant virus using gold nanoparticle-modified electrodes. Anal Chim Acta.

[41] Rezaei N, Moshaii A, Safarnejad MR, Sajedi RH, Rahmanipour M (2025). Attomolar electrochemical direct and sandwich immunoassays for the ultrasensitive detection of tomato brown rugose fruit virus. Plant Methods.

[42] Hemmer C, Djennane S, Ackerer L, Hleibieh K, Marmonier A (2018). Nanobody-mediated resistance to grapevine fanleaf virus in plants. Plant Biotechnol J.

[43] Buja I, Sabella E, Monteduro AG, Rizzato S, Bellis LD (2022). Detection of *Ampelovirus* and *Nepovirus* by Lab-on-a-Chip: a promising alternative to ELISA test for large scale health screening of grapevine. Biosensors.

[44] Zhang J, Sun H, Pei W, Jiang H, Chen J (2021). Nanobody-based immunosensing methods for safeguarding public health. J Biomed Res.

[45] Sahu S, Dietrich S, Boukherroub R, Poignavent V, Trolet A Unleashing the power of double-nanobody sandwich electrochemical assay for the rapid detection of grapevine fanleaf virus. *SSRN*.

[46] Orlov I, Hemmer C, Ackerer L, Lorber B, Ghannam A (2020). Structural basis of nanobody recognition of grapevine fanleaf virus and of virus resistance loss. Proc Natl Acad Sci USA.

[47] Sadighbayan D, Hasanzadeh M, Ghafar-Zadeh E (2020). Biosensing based on field-effect transistors (FET): Recent progress and challenges. TrAC-Trend Anal Chem.

[48] Poghossian A, Jablonski M, Molinnus D, Wege C, Schöning MJ (2020). Field-effect sensors for virus detection: from ebola to SARS-CoV-2 and plant viral enhancers. Front Plant Sci.

[49] Jablonski M, Poghossian A, Keusgen M, Wege C, Schöning MJ (2021). Detection of plant virus particles with a capacitive field-effect sensor. Anal Bioanal Chem.

[50] Berto M, Vecchi E, Baiamonte L, Condò C, Sensi M (2019). Label free detection of plant viruses with organic transistor biosensors. Sens Actuators B Chem.

[51] Han S, Pasquini D, Sorieul M, Boratto MH, Gatecliff L (2025). Implantable ion‐selective organic electrochemical transistors enable continuous, long‐term, and in vivo plant monitoring. Advanced Science.

[52] Peng Y, Dallas MM, Ascencio-Ibáñez JT, Hoyer JS, Legg J (2022). Early detection of plant virus infection using multispectral imaging and spatial–spectral machine learning. Sci Rep.

[53] Petkidis A, Andriasyan V, Murer L, Volle R, Greber UF (2024). A versatile automated pipeline for quantifying virus infectivity by label-free light microscopy and artificial intelligence. Nat Commun.

[54] Ning H, Boyes I, Numanagić I, Rott M, Xing L (2024). Diagnostics of viral infections using high-throughput genome sequencing data. Brief Bioinform.

[55] Maina S, Donovan NJ, Plett K, Bogema D, Rodoni BC (2024). High-throughput sequencing for plant virology diagnostics and its potential in plant health certification. Front Hortic.

[56] Villamor DEV, Ho T, Al Rwahnih M, Martin RR, Tzanetakis IE (2019). High throughput sequencing for plant virus detection and discovery. Phytopathology.

[57] Massart S, Olmos A, Jijakli H, Candresse T (2014). Current impact and future directions of high throughput sequencing in plant virus diagnostics. Virus Res.

[58] Javaran VJ, Poursalavati A, Lemoyne P, Ste-Croix DT, Moffett P (2023). NanoViromics: long-read sequencing of dsRNA for plant virus and viroid rapid detection. Front Microbiol.

[59] Mongan AE, Tuda JSB, Runtuwene LR (2020). Portable sequencer in the fight against infectious disease. J Hum Genet.

[60] Ramos-Mandujano G, Grünberg R, Zhang Y, Bi C, Guzmán-Vega FJ (2023). An open-source, automated, and cost-effective platform for COVID-19 diagnosis and rapid portable genomic surveillance using nanopore sequencing. Sci Rep.

[61] Ji P, Aw TG, Van Bonn W, Rose JB (2020). Evaluation of a portable nanopore-based sequencer for detection of viruses in water. J Virol Methods.

[62] Szunerits S, Pagneux Q, M’Barek YB, Vassal S, Boukherroub R (2023). Do not let electrode fouling be the enemy of bioanalysis. Bioelectrochemistry.

[63] Russo MJ, Han M, Desroches PE, Manasa CS, Dennaoui J (2021). Antifouling strategies for electrochemical biosensing: mechanisms and performance toward point of care based diagnostic applications. ACS Sens.

[64] Mohammad A, Eleyan D, Eleyan A, Bejaoui T (2024). IoT-Based Plant Disease Detection Using Machine Learning: A Systematic Literature Review.

[65] Boykin LM, Sseruwagi P, Alicai T, Ateka E, Mohammed IU (2019). Tree lab: portable genomics for early detection of plant viruses and pests in sub-saharan Africa. Genes.

[66] Charlermroj R, Himananto O, Seepiban C, Kumpoosiri M, Warin N (2013). Multiplex detection of plant pathogens using a microsphere immunoassay technology. PLoS One.

[67] Pan M, Yang J, Liu K, Yin Z, Ma T (2020). Noble metal nanostructured materials for chemical and biosensing systems. *Nanomaterials*.

[68] Kanapiya A, Amanbayeva U, Tulegenova Z, Abash A, Zhangazin S (2024). Recent advances and challenges in plant viral diagnostics. Front Plant Sci.

